# Supplementary material for: Non-interferometric stand-alone single-shot holographic camera using reciprocal diffractive imaging
Source: Nat Commun. 2023 Aug 12;14:4870. doi: 10.1038/s41467-023-40019-0 (PMC10423261; doi:10.1038/s41467-023-40019-0)
Supplement: Supplementary file 2 — Description of Additional Supplementary Files [file 41467_2023_40019_MOESM2_ESM.pdf]

Description of Additional Supplementary Files

Supplementary Video 1:

Results for the numerical refocusing of the dice in Fig. 3 from -300 to +300 mm. The field of view is 24.75 × 24.75 mm^2.

Supplementary Video 2:

Real-time imaging of the letters "AB" in Fig. 4c. The letters "AB" were imaged for 16.47 at a frame rate of 4.25 fps. The field of view is 20.625 × 20.625 mm^2.

Supplementary Video 3:

Real-time imaging of the rotating 10 × 10 × 10 mm^3 dice in Fig. 4d. The dice were imaged for 22.35 s at a frame rate of 4.25 fps. The field of view is 28.16 × 28.16 mm^2.

Supplementary Video 4:

Animation of the moving dolls in Fig. 5. A total of 193 scenes were captured within 2 min. The field of view is 28.16 × 28.16 mm^2.
